# Supplementary material for: The genome sequence of a soldier beetle, Rhagonycha lutea (Müller, 1764)
Source: Wellcome Open Res. 2025 Jan 15;10:24. [Version 1] doi: 10.12688/wellcomeopenres.23467.1 (PMC12859421; doi:10.12688/wellcomeopenres.23467.1)
Supplement: Peer review history

# Reviewer report: Reviewer response for version 1

**DOI:** 10.21956/wellcomeopenres.25877.r125155

**Published:** 2026-01-30

**Authors:** Hermes E. Escalona

**Affiliations:** 1 Australian National Insect Collection, CSIRO, Australia

The article is well written and provides detailed preliminary information on the distribution, biology and taxonomy of the species *Rhagonycha lutea. *The methodology is concise with adequate references and relevant details. The genome quality seems high with an M50 of 71Mb, the number of annotated proteins is within the average for beetles. The pipeline for screening for contamination is provided in a link.

 I recommend the publication of this article.

 Pl correct:

 "(soldier beetle; Arthropoda; Insecta; Coleoptera; Cantharidae)" to

 (Insecta; Coleoptera; Cantharidae)

 "Genome notes" to

 The genomes of seven species of the same .....

Are sufficient details of methods and materials provided to allow replication by others?

Yes

Is the rationale for creating the dataset(s) clearly described?

Yes

Are the datasets clearly presented in a useable and accessible format?

Yes

Are the protocols appropriate and is the work technically sound?

Yes

Reviewer Expertise:

Entomology

I confirm that I have read this submission and believe that I have an appropriate level of expertise to confirm that it is of an acceptable scientific standard.

# Reviewer report: Reviewer response for version 1

**DOI:** 10.21956/wellcomeopenres.25877.r125156

**Published:** 2025-07-14

**Authors:** Christopher B. Cunningham

**Affiliations:** 1 University of Georgia, Georgia, USA

The project continues to produce quality resources for the insect genomics community. The methods are sound and relatively well documented. The article needs has enough details to be reasonably reproducible. The data supporting the publication are publicly available.

 Abstract. There is no justification within the abstract, just results. At least a sentence that this was part of a large survey of insects is needed. And one sentence for its possible value.

 Keywords. None given.

 Background. Background. The stated motivation for this study in the introduction is fine – part of a very large survey. However, it would be nice for the reader to understand if this is the first of its genus, is of particular interest for ecological or genetics studies, or something unusual about its biology. Helping the reader understand what this genome might help with investigation in. Almost all the copious details of its life history and biogeography are irrelevant to the reader; not wrong, just not needed to understand this work. The paper is not about its ecology or morphology, but about its genetics.

 Results. Table 1 is not really needed. They are not details that the reader needs to under the work and they can all be found under the NCBI BioProject ID or are repeated from the methods paragraph.

 There is a large amount of duplications of BUSCO for a haplotype purged primary assembly, close to 4%. That is about double the most that I would expect from a true primary haplotype assembly. This needs to be further assessed to make sure duplications are real and are not interfering with downstream analyses.

 Figure 3. That is a very odd blob plot. The no-hit category is almost 10% of the assembly and is highly constrained to a specific GC content. I double it, but is that the sex chromosome? It's all non-scaffolded stuff too. It is such as an extreme break from the scaffolds. That needs further investigation before it should be accepted as part of the assembly.

 BUSCO completeness needs to be assessed with the updated OrthoDB version 12. Difference from 10 to 12 have made a big difference for several insects I work with, and colleagues have reported the same. The paper should not be indexed until this is updated. The new version has been for 6 months.

 Figure 4. Fig 4 has very little information beyond what is found elsewhere in the manuscript and can easily be summarized as a sentence in the results.

 That is a huge number of genes for a beetle (or any insect really). Tribolium has 12.1K protein coding. I see that others of this family have this high gene counts, but from the same annotation pipeline. What QC was done? No BUSCO of the annotation was given. It's not something a reader can accept without more justification. I understand that it's from a standardized pipeline, but sometimes otherwise reasonable defaults produce poor results for lesser-studied species/groups. Either the annotation needs to be justified more robustly, or this should be removed from the manuscript. The link to the annotation is also dead. ENSEMBL is no longer releasing Rapid Releases apparently.  

 Minor Comments.

 Species names at the beginning of a sentence should have the genus spelled out.

Are sufficient details of methods and materials provided to allow replication by others?

Yes

Is the rationale for creating the dataset(s) clearly described?

Partly

Are the datasets clearly presented in a useable and accessible format?

Yes

Are the protocols appropriate and is the work technically sound?

Partly

Reviewer Expertise:

Genetics, Genomics, Epigenetics, Behavior, Reproduction, Insects

I confirm that I have read this submission and believe that I have an appropriate level of expertise to state that I do not consider it to be of an acceptable scientific standard, for reasons outlined above.

# Reviewer report: Reviewer response for version 1

**DOI:** 10.21956/wellcomeopenres.25877.r117371

**Published:** 2025-01-21

**Authors:** Ruiqi Li

**Affiliations:** 1 University of Colorado Boulder, Boulder, Colorado, USA

Barclay et al. presents a chromosome-level genome sequence for *Rhagonycha lutea. *The genome is of high-quality and will provide great genetic resources for the global scientific community. I only have a few minor comments for the introduction.

 1. online NBN map: what does NBN stand for? 

 2. I like the authors present some basic ecological information of the species in the introduction. However, could the authors add another paragraph on what questions this genome could help us answer? Any interesting traits? Is there any interesting phylogenetic questions about the species? You can refer to Li et al. 2024, and Carter et al. 2020.

Are sufficient details of methods and materials provided to allow replication by others?

Yes

Is the rationale for creating the dataset(s) clearly described?

Yes

Are the datasets clearly presented in a useable and accessible format?

Yes

Are the protocols appropriate and is the work technically sound?

Yes

Reviewer Expertise:

Genomics

I confirm that I have read this submission and believe that I have an appropriate level of expertise to confirm that it is of an acceptable scientific standard.

## References

[1] : The genome sequence of the smooth giant clam, Tridacna derasa Röding, 1798. *Wellcome Open Res* .2024;9: 10.12688/wellcomeopenres.22618.1 375 10.12688/wellcomeopenres.22618.1 39429632 PMC11490831

[2] : Complete mitochondrial genomes provide current refined phylogenomic hypotheses for relationships among ten Hirundo species. *Mitochondrial DNA B Resour* .2020;5(3) : 10.1080/23802359.2020.1790999 2881-2885 10.1080/23802359.2020.1790999 33457987 PMC7783031
